# Quantitative determination of the interaction potential between two surfaces using frequency-modulated atomic force microscopy

**DOI:** 10.3762/bjnano.11.60

**Published:** 2020-05-06

**Authors:** Nicholas Chan, Carrie Lin, Tevis Jacobs, Robert W Carpick, Philip Egberts

**Affiliations:** 1 Department of Mechanical and Manufacturing Engineering, University of Calgary, 40 Research Place NW, Calgary, Alberta T2L 1Y6, Canada; 2 Department of Mechanical Engineering and Materials Science, University of Pittsburgh, Pittsburgh, PA 15621, USA; 3 Department of Mechanical Engineering and Applied Mechanics, University of Pennsylvania, 220 S. 33rd Street, Philadelphia, Pennsylvania 19104, USA

**Keywords:** adhesion, atomic force microscopy, diamond, frequency-modulated AFM, interaction potential, Lennard-Jones, surfaces

## Abstract

The interaction potential between two surfaces determines the adhesive and repulsive forces between them. It also determines interfacial properties, such as adhesion and friction, and is a key input into mechanics models and atomistic simulations of contacts. We have developed a novel methodology to experimentally determine interaction potential parameters, given a particular potential form, using frequency-modulated atomic force microscopy (AFM). Furthermore, this technique can be extended to the experimental verification of potential forms for any given material pair. Specifically, interaction forces are determined between an AFM tip apex and a nominally flat substrate using dynamic force spectroscopy measurements in an ultrahigh vacuum (UHV) environment. The tip geometry, which is initially unknown and potentially irregularly shaped, is determined using transmission electron microscopy (TEM) imaging. It is then used to generate theoretical interaction force–displacement relations, which are then compared to experimental results. The method is demonstrated here using a silicon AFM probe with its native oxide and a diamond sample. Assuming the 6-12 Lennard-Jones potential form, best-fit values for the work of adhesion (*W*_adh_) and range of adhesion (*z*_0_) parameters were determined to be 80 ± 20 mJ/m^2^ and 0.6 ± 0.2 nm, respectively. Furthermore, the shape of the experimentally extracted force curves was shown to deviate from that calculated using the 6-12 Lennard-Jones potential, having weaker attraction at larger tip–sample separation distances and weaker repulsion at smaller tip–sample separation distances. This methodology represents the first experimental technique in which material interaction potential parameters were verified over a range of tip–sample separation distances for a tip apex of arbitrary geometry.

## Introduction

Knowledge of material interface interaction behavior is crucial to the design of nanometer-scale devices and processes, such as high-density hard disk storage [1], digital light processing (DLP) projectors [2–3], atomic force microscopy (AFM) [4–5], and nanolithography techniques [6]. In particular, material parameters, such as interfacial adhesion, friction and wear (in the case of translating surfaces), significantly impact the success of the aforementioned examples. For instance, micromirrors, present in DLP technology, require coatings to prevent sticking of the mirrors due to relatively high adhesive forces [3]. Therefore, there has been considerable research into surface modification (e.g., lubricants [7–9], surface functionalization [10–11], and surface texturing [12–14]) in order to mitigate the impact of such issues. For example, the adsorption of self-assembled monolayers on contacting surfaces is one method by which the surface can be modified to reduce the detrimental impacts of adhesion, friction and wear [15–17]. The nanometer length scales over which these processes modify surface interactions necessitates characterization methods with angstrom-level precision. Therefore, techniques such as atomic force microscopy (AFM) are often used to evaluate the performance of these surface modification approaches.

The interpretation of lubrication and surface modification behavior via AFM experiments is often challenging as the measured adhesion and friction forces cannot provide direct mechanistic understanding of atomic-scale interactions that occur at the contact interface. Thus, it is often desirable to incorporate complementary atomistic simulation techniques [18–21] or contact mechanics models [22–25] to allow better visualization of surface interactions. While useful insights can be obtained using fully atomistic simulations, such as molecular dynamics simulations or density functional theory, these techniques are impractical for describing larger contacts with a large number of atoms. In these cases of larger bodies, contact modeling is used, where the interaction forces are computed by numerically integrating a surface potential [22]. In such cases, the Lennard-Jones (LJ) potential is commonly used [22,26–27]. Furthermore, given the difficulty of accurately representing interactions between mismatched materials, many simulations in tribology and interfacial science also use the LJ potential or potentials based on it [27–31]. However, there is little direct experimental validation of this numerical integration approach. Here, we demonstrate a novel method for directly comparing experimentally measured interaction forces, via AFM, with theoretical pair potentials for a given material system. By doing so, we are able to determine the work and range of adhesion (*W*_adh_ and *z*_0_, respectively) material parameters, assuming a given pair potential. Additionally, the method can be extended to the evaluation of pair potential forms for a given material system. In this study, we use the methodology to compare our experimental results with the well-known 6-12 LJ pair potential. This potential was originally developed as a mechanism to understand and interpret the interaction of inert gas atoms/molecules assumed to be point particles [32]. Despite the origins of the potential function, it has also shown success in replicating the interactions observed in experiments regarding solid bodies [18,33].

The 6-12 LJ pair potential can be extended to larger-scale bodies through the integration of the potential across a given geometry [34–35]. This allows for the prediction of interaction forces between irregularly shaped solid bodies. Alongside this, advances in the field of AFM have allowed for precise measurements of interaction forces, as well as the control of environment and probe/sample materials [36–37]. Therefore, we seek to leverage these advances to create a methodology for better validation of empirical pair potentials. In particular, we will do this by combining measurements of tip–sample interaction forces and experimental tip apex geometries.

In this manuscript, frequency modulation (FM) AFM was used to determine the interaction forces between two irregularly shaped solids: the apex of a silicon AFM probe with its native oxide and a slightly roughened, nominally flat single-crystal diamond surface. This substrate was chosen due to its high mechanical stiffness, chemical inertness and stability, and interest for tribological applications [38]. It also is a good representation for other hard engineering materials and coatings, including having a low but finite surface roughness, as specified below. Spectroscopy measurements were performed where the change in fundamental flexural resonance of the AFM probe was measured as a function of the relative piezoactuator displacement. These measurements will be referred to as Δ*f*–*d* curves for the remainder of the manuscript. Δ*f*–*d* curves were used to extract a relation between tip–sample interaction forces and relative tip–sample separation displacement, referred to as piezoactuator displacement henceforth. This relation will be referred to as *F*(*z*) curves for the remainder of the manuscript. Experimental *F*(*z*) curves were then compared with theoretical LJ *F*(*z*) curves. These LJ *F*(*z*) curves were generated using the 6-12 LJ pair potential form and experimental tip apex geometry, which was extracted from two-dimensional transmission electron microscopy (TEM) images. In prior works, the shapes of AFM probes have been determined using field ion microscopy (FIM) [39], atom probe tomography (APT) [40] and transmission electron microscopy (TEM) [35]. Each technique has strengths and weaknesses: FIM has can resolve the exact atomic structure of the tip apex, but only for conductive tips; APT combines FIM with atom identification, but is a destructive technique; and TEM has less restrictions on the tip material and can provide atomic-level resolution, but only images the tip in a profile view. For the present investigation, TEM was chosen because of the ease of sample preparation and the ability to image not only the tip apex with atomic resolution, but also the shape of the tip shank that contributes to the long-range interaction between tip and sample. Best-fit potential parameters were determined for the silicon oxide–diamond system and the spatial variance of these parameters was examined over different locations across the diamond sample.

## Experimental

### Frequency modulation atomic force microscopy

FM-AFM is a mode of AFM that allows for the probing of tip–sample interaction forces with the possibility of atomic resolution [41]. In this method, the probe is oscillated at its fundamental flexural resonance frequency (i.e., normal to the sample) and at a constant amplitude, while it is scanned laterally relative to the sample. A piezoactuator acting in the *z*-direction brings the probe closer or further from the sample. Due to non-linear tip–sample interaction forces, the resonance frequency of the oscillating cantilever will shift. This shift can be used as a feedback signal to measure the sample topography, among other parameters. In order to determine the interaction force behavior as a function of the separation distance, we measured the frequency shift of the oscillating cantilever as a function of the separation distance (Δ*f*–*d* curves) between a silicon AFM probe and a diamond sample. An analytical relationship between the resonance frequency shift and the tip–sample interaction force in FM-AFM was first derived by Giessibl [42] and is seen in the following equation:

[1] $$\frac{\Delta f}{f_{\text{res}}}=-\frac{1}{\pi ak}\int_{-1}^{1}F\left(z+a\left(1+u\right)\right)\frac{u}{\sqrt{1-u^{2}}}\text{d}u\;.$$

In Equation 1, Δ*f* represents the change in the primary flexural resonance frequency of the cantilever near the surface, *f*_res_ represents the primary flexural resonance frequency of the cantilever far from the surface, *k* represents the probe stiffness, *a* represents the amplitude of oscillation, *F* represents the tip–sample interaction force, and *z* represents the smallest separation distance between the tip and sample during oscillation.

While this equation is useful for determining cantilever resonance frequency changes from a known tip–sample interaction force, this equation becomes unwieldy when trying to determine tip–sample interaction forces from changes in the normal resonance frequency of the cantilever. Fortunately, several solutions to the inversion of Equation 1 have been determined, first for specific ranges of oscillation amplitudes [43–44] and later for arbitrary oscillation amplitudes [45–46]. In our case, we used the equations outlined in [45] for arbitrary amplitudes to directly calculate tip–sample interaction forces:

[2] $$F\left(z\right)=2k\int_{z}^{2}\left(1+\frac{a^{1/2}}{8\sqrt{\pi \left(t-z\right)}}\right)\Omega \left(t\right)-\frac{a^{3/2}}{\sqrt{2\left(t-z\right)}}\frac{\text{d}\Omega \left(t\right)}{\text{d}t}\text{d}t\;.$$

In Equation 2, Ω(*z*) = Δ*f*(*z*)/*f*_res_. From Equation 2, we can extrapolate the interaction force and potential curves as a function of the tip–sample separation distance from an experimental Δ*f*–*d* curve for a chosen material system using FM-AFM. From this, we can determine *W*_adh_ and *z*_0_ by matching these *F*(*z*) curves to a set of LJ *F*(*z*) curves generated for the specific tip apex shape, as described in the following section.

### Determination of work and range of adhesion using in situ TEM adhesive experiments

**Figure 1:**
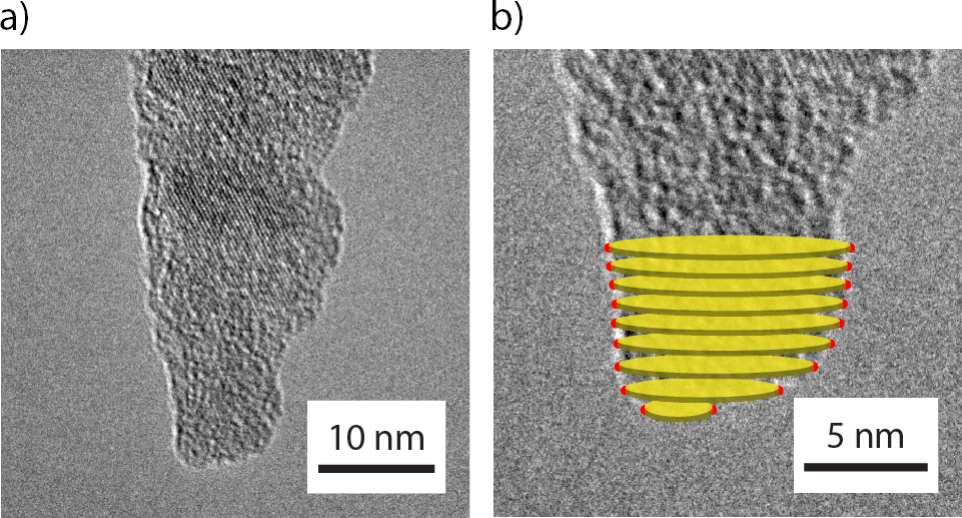
(a) TEM image of the apex of the AFM tip used in FM-AFM measurements. (b) Magnified TEM image of the AFM tip shown in Figure 1a. The thin yellow disks represent the methodology by which the two-dimensional tip apex profile is turned into a three-dimensional tip apex volume.

The method that will be discussed to determine LJ parameters from dynamic FM-AFM measurements is extended from the experimental method proposed by Jacobs et al. [35,47], which is based on static force–displacement measurements. The procedure is as follows: In situ force–separation measurements between an AFM probe and a nominally flat diamond punch are performed in a TEM, where the pull-off force and the snap-in distance are measured. The use of in situ TEM allows for a direct characterization of the AFM tip shape. Furthermore, TEM allows for measurements of probe deflection, which can be converted to the normal force using the calibrated normal force constant of the cantilever and the separation distance between the tip and substrate continuously as the sample is displaced. The two-dimensional profile of the tip apex geometry was obtained through simultaneously acquired TEM images during experiments (Figure 1a) and transformed into a three-dimensional tip structure by using the method of disks, whereby the tip is approximated as a stack of thin axisymmetric disks normal to the flat diamond surface (Figure 1b). Using these additional data, an LJ *F*(*z*) curve was generated by assuming that the silicon oxide–carbon interaction is described by the 6-12 LJ pair potential. This was achieved by using the following equation:

[3] $$\sigma_{\text{normal}}\left(z\right)=\frac{F_{\text{surf}}}{A_{\text{surf}}}=-\frac{8W_{\text{adh}\text{,int}}}{3z_{0}}\left({\left(\frac{z_{0}}{z}\right)}^{3}-{\left(\frac{z_{0}}{z}\right)}^{9}\right)\;.$$

Equation 3 refers to the surface-traction equation that describes the normal stress (σ_normal_) , or the surface force *F*_surf_ per surface area *A*_surf_, associated with two parallel half spaces, separated by a static distance *z*. This is contrasted to the distance *z* shown in Equation 2, which represents the smallest separation distance between the tip and the sample during oscillation. In this case, the slight difference in definition can be disregarded as the interaction force determined using Equation 2 is always calculated at the same relative point during an oscillation cycle of the AFM probe. In this study, a theoretical tip–sample interaction force was calculated for a range of tip–sample separation distances via the integration of Equation 3 across the whole surface area of the generated tip apex geometry, while considering the diamond surface as an ideal flat half-space. This procedure results in a theoretical LJ *F*(*z*) curve unique to the geometry of the tip used in the study.

Multiple LJ *F*(*z*) curves were generated for a range of *W*_adh_ and *z*_0_ values and compared to experimental measurements of pull-off forces and snap-in distances to determine best-fit parameters for the particular material system.

### Experimental details

All experiments were conducted in a RHK 750 UHV-AFM system operated under ultrahigh vacuum (UHV) conditions (1 × 10^−10^ Torr) and at room temperature. Single-crystal diamond(100) samples (EDP Corporation) were cleaned in an ultrasonic bath with acetone and subsequently ethanol for 20 min with each solvent. The samples were then transferred into the vacuum chamber and baked at 120 °C for 8 h.

A non-reflective silicon AFM probe (PPP-NCL, Nanosensors) was used in AFM measurements. TEM images were taken of the AFM tip apex before mounting the probe in an AFM probe holder and transferring it into the UHV AFM. The tip apex was imaged using a custom TEM holder, described in [48], in a JEOL 2010F TEM. In all TEM images, rotation of the AFM tip axis was required to account for tilting of the cantilever body within the TEM image, as well as the 22.5° angle that is imposed on the cantilever from the AFM probe holder. The first factor is accounted for by taking low-magnification TEM images, where the main cantilever body was visible in the TEM images, followed by increasingly higher magnification TEM images to obtain a high-resolution profile of the tip apex. The images were then stitched together to determine the necessary rotation required to account for the tilt caused by the TEM holder. The second factor was accounted for by rotating the TEM images by an additional 22.5°.

Following TEM imaging, the probe was mounted to an AFM probe holder, transferred into the vacuum chamber, and baked at 120 °C for 1 h to remove any residual adsorbed moisture and other adsorbates on the probe. No tip preparation beyond this was conducted, to ensure that the tip structure remained as similar to the initial images and as small as possible. Before topographic imaging of the surface, the tip–sample contact potential difference was determined by measuring the probe frequency shift as a function of the sample bias voltage. A DC bias was then applied to the sample surface for all subsequent measurements to compensate for this potential difference. Initial topographic images of the diamond(100) surface were obtained before performing Δ*f*–*d* curve measurements. Following topographic imaging of the surface, Δ*f*–*d* curves were acquired on a 8 × 8 grid over a 500 nm × 500 nm scan area, with 20 curves acquired in each cell of the grid. Following these measurements, the oscillation amplitude of the cantilever was calibrated using the procedure described in [49]. The spring constant of the cantilever was calculated using the beam geometry method [50], where the fundamental flexural resonance frequency was measured and used to determine the thickness of the cantilever. The length and width of the cantilever were measured using optical microscopy. In this study, the normal bending spring constant of the cantilever was determined to be 30.5 N/m with an approximate uncertainty of 10% based on the cantilever dimensions. In this study, the oscillation amplitude was set to be 12 ± 1 nm (the error resulting from the calibration of the oscillation amplitude, which is primarily impacted by the uncertainty in the cantilever dimensions, rather than a variance in the amplitude over the course of the measurement). Other cantilevers used in this study were calibrated similarly where the oscillation amplitude varied from 8.6 to 12.1 nm. Directly following the acquisition of the Δ*f*–*d* curves, the probe was removed from the vacuum chamber and post-mortem TEM images of the tip apex were acquired.

## Results

**Figure 2:**
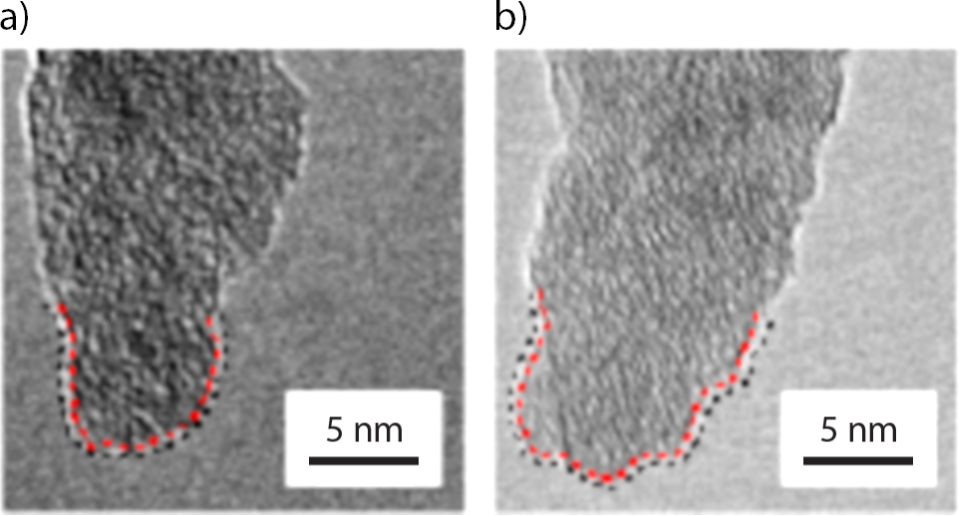
(a) TEM image of the AFM tip apex before AFM imaging. (b) TEM image of the AFM tip apex after AFM imaging. No appreciable change was observed in tip apex radius when comparing Figure 2a and Figure 2b. Furthermore, an ordered silicon lattice structure is observed in both Figure 2a and Figure 2b. The absence of perturbations in the silicon lattice suggests plastic deformation of the AFM tip did not occur during the AFM experiments. Rotation of the tip apex is apparent between TEM imaging before and after AFM experiments. This is primarily caused by residual glue on the cantilever chip after AFM measurements. In both (a) and (b), a black dotted line shows the traced tip apex while the red dotted line shows the interface between SiO*_x_* and Si. No change in the distance between the SiO*_x_* and the crystalline Si is observed between tip (a) and (b).

Figure 2a shows the initial shape of the tip apex used, and Figure 2b shows its shape after the AFM experiments. The spatial resolution of the TEM is of the order of several angstroms, as observed by the resolved silicon crystal lattice shown in Figure 1a, as well as in Figure 2a and Figure 2b. Qualitatively, we observe no appreciable change in the tip apex radius between tip images before and after AFM imaging. Additionally, we observe similar ordering in crystal lattices and an absence of defects in the lattice fringes for both figures. These observations suggest that plastic deformation did not occur during AFM experiments. It is also clear that Figure 2a and Figure 2b appear different as a result of the angle variation when mounting the probe into the TEM holder. This variation is exacerbated by residual glue that is present on the cantilever chip following the experiments. These factors prevent reproducible orientation of the tip apex when acquiring TEM images before and after AFM experiments. Due to this angle variation, we generate the three-dimensional tip volume of the AFM tip apex from TEM images taken before AFM imaging.

**Figure 3:**
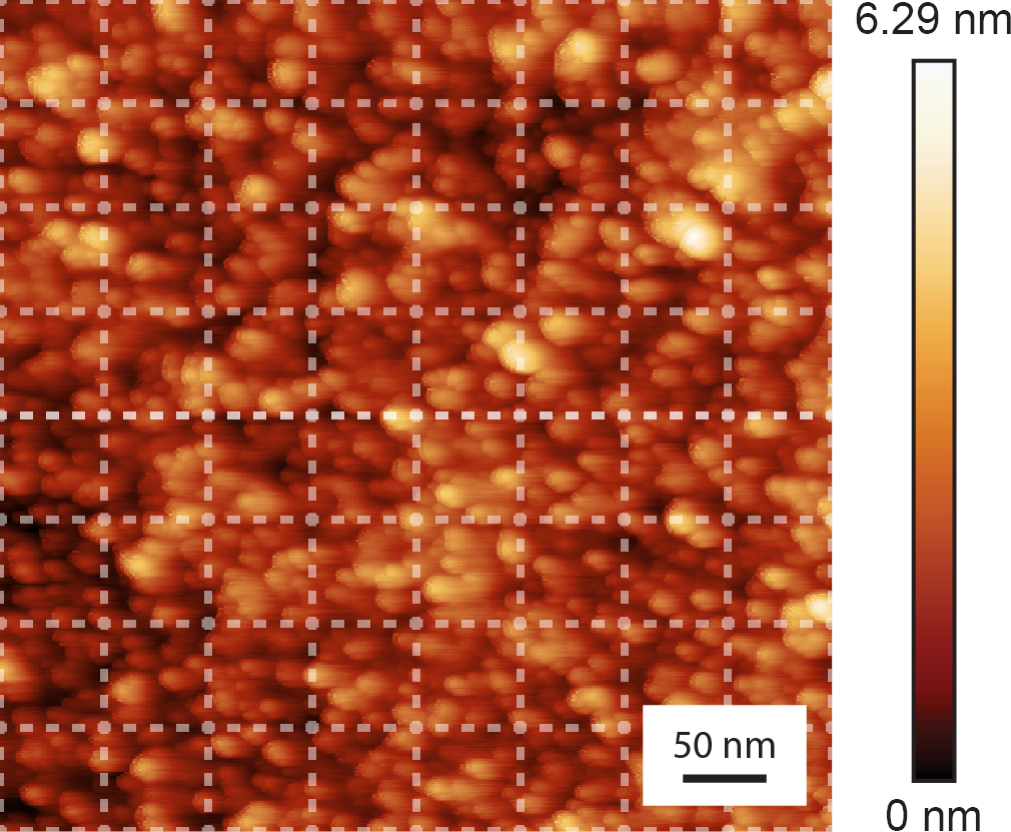
Topographic AFM image of the diamond surface showing the nanometer-scale roughness of the surface. Dashed lines show the grid on which the Δ*f*–*d* curves were acquired.

Figure 3 shows a 500 nm × 500 nm topographic AFM image of the diamond surface, from which the subsequent Δ*f*–*d* curves are taken. The surface shows topographical roughness on the crystal. However, the calculated height variation and RMS roughness are relatively low, being 3.90 nm and 0.78 nm, respectively. When acquiring the subsequent force–distance curves, the surface was divided into an equally spaced 8 × 8 grid (62.5 nm wide squares), which is represented by the white dashed-line grid overlaid on the topographic image. 20 Δ*f*–*d* curves are taken at the center of each square.

**Figure 4:**
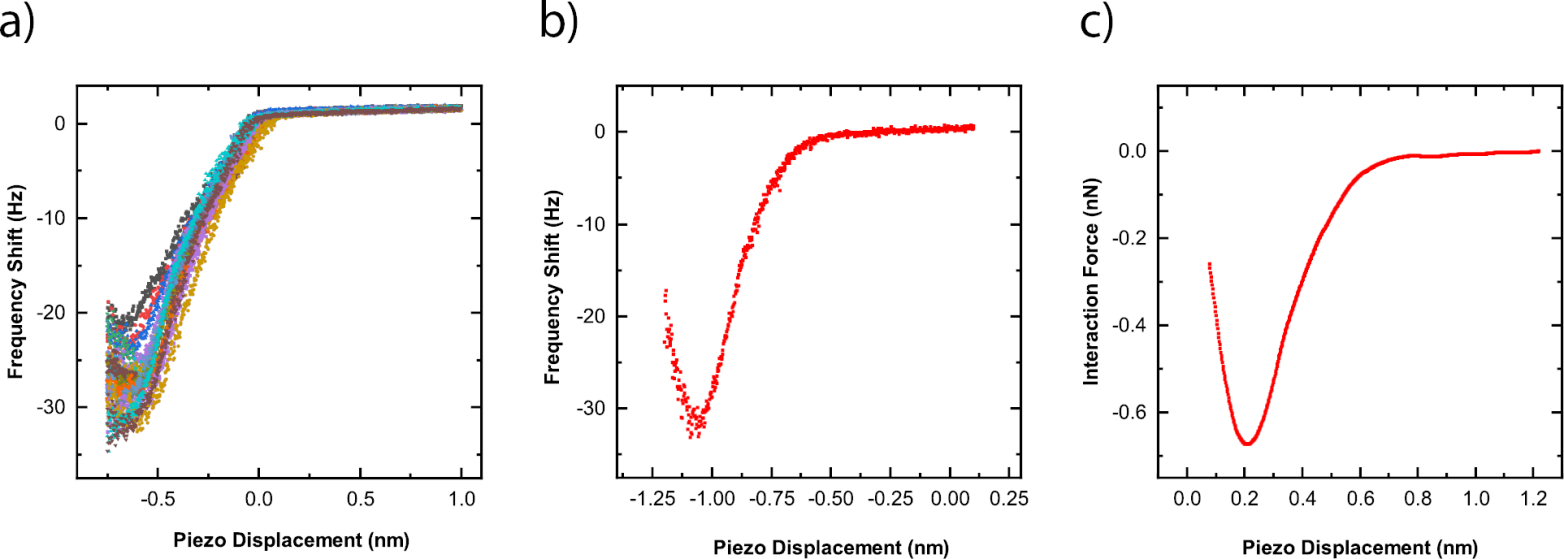
(a) 20 Δ*f*–*d* curves measured at one grid position on the diamond sample using a silicon AFM probe. (b) An example of a single Δ*f*–*d* curve from the same grid position as in Figure 5a. (c) The corresponding calculated interaction force from Figure 5b, as calculated via the method developed by Sader and Jarvis [45].

Figure 4a shows the 20 Δ*f*–*d* approach curves acquired from one of the squares in the grid shown in Figure 3. In the subsequent analysis, only the approach curves were considered because the retraction curves have been previously shown to be affected by creep of the piezoelectric actuator and energy dissipation processes [51–52]. These artifacts and dissipation processes are indistinguishable from the interaction forces, rendering the retraction curves far more difficult to quantitatively analyze. For this reason, the interaction potential is assumed to be identical upon approach and retraction. Comparable curves were acquired with the other tips used in this study. While there is some variation in the Δ*f*–*d* curves, we observed a non-monotonic trend in all measurements. As the probe starts approaching the sample surface, a decrease in the frequency shift is observed. At some point, a minimum in frequency shift is reached. Then, the frequency shift continually increases as tip–sample separation distance continues to decrease. This decreasing and increasing trend in the frequency shift is indicative of the competing attractive and repulsive interaction forces between the probe and the sample, where attractive forces are more dominant at larger tip–sample separation distances, and repulsive forces are more dominant at shorter tip–sample separation distances. The *x*-axis for each curve is shifted to compensate for the drift of the piezoactuator between each Δ*f*–*d* measurement. Figure 4b and Figure 4c show a single exemplary Δ*f*–*d* curve at the same position and the corresponding calculated interaction force, respectively. Each Δ*f*–*d* curve measured was converted into an *F*(*z*) curve using the procedure of Sader and Jarvis [45]. It should be noted that there is a smoothing algorithm applied to the Δ*f*–*d* curve to prevent spurious force jumps caused by noise in the Δ*f*–*d* curves when converted to *F*(*z*) curves. Furthermore, we assumed that the interaction force relation for the silicon oxide–carbon system is a smooth function with a single localized force minimum. Therefore, several extracted *F*(*z*) curves were disregarded due to multiple localized minima in the force relation. This resulted in four locations in the 8 × 8 grid experiment being disregarded.

LJ *F*(*z*) curves, incorporating the experimental tip shape, were generated using the potential algorithm outlined in [53], for which *W*_adh_ and *z*_0_ were varied between 0.01 and 150 J/m^2^ and between 0.1 and 3 nm, respectively. The discretization of the tip–sample separation distance for the LJ *F*(*z*) curves was chosen to match the spacing of the piezoactuator displacement between data points in experiments. This discretization eliminates the need for interpolation when performing the regression analysis described below.

Information on the absolute tip–sample separation distance is not included in the experimental Δ*f*–*d* curves or in the subsequently calculated *F*(*z*) curves. Rather, piezoactuator displacement in these measurements refers to relative position of the piezoactuator that is attached to the end of the AFM probe, opposite to the tip. Therefore, there is significant difficulty comparing FM-AFM data to *F*(*z*) curves generated via theoretical pair potentials. To quantitatively determine the tip–sample separation distance from the experimental measurements, a least squares regression analysis was performed to best align the converted experimental *F*(*z*) curves with each LJ *F*(*z*) curve. Following this, best-fit parameters for *W*_adh_ and *z*_0_ were determined by searching for an LJ *F*(*z*) curve with adhesion parameters that best minimized the squared residual from the regression analysis.

**Figure 5:**
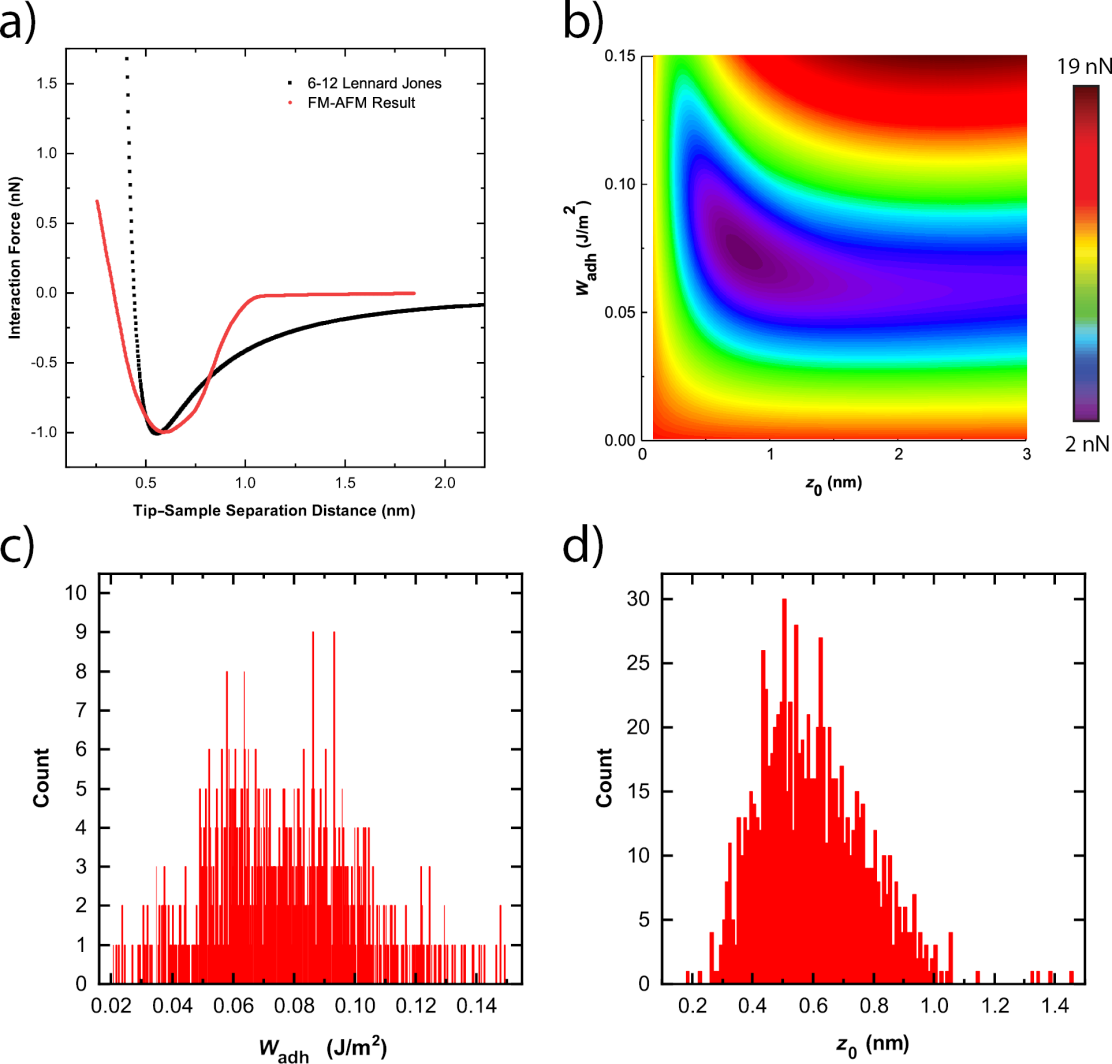
(a) Example of an experimentally derived interaction force–distance curve compared to a theoretically derived force–distance curve with best-fit parameters for *W*_adh_ and *z*_0_. Theoretical LJ *F*(*z*) curves were calculated using a tip apex profile generated by TEM images taken before the AFM experiments. In this case, the best-fit parameters for *W*_adh_ and *z*_0_ are 0.0860 J/m^2^ and 0.55 nm, respectively. (b) An example of a square-rooted variance map produced using a least squares fitting technique to compare the experimental *F*(*z*) curves to LJ *F*(*z*) curves. (c, d) Histograms of the best-fit *W*_adh_ and *z*_0_ values, respectively, over the entire diamond surface.

Figure 5a shows a typical result using the aforementioned least squares regression analysis to systematically compare an LJ *F*(*z*) curve with an experimentally extracted *F*(*z*) curve. Figure 5b shows the square-rooted variance map associated with the parametric search of the best-fit *W*_adh_ and *z*_0_ values. The least squares regression analysis clearly shows that the parametric search produces a singular minimum for best-fit *W*_adh_ and *z*_0_ values.

## Discussion

Figure 5c shows the histogram plot for the best-fit *W*_adh_ values over the entire diamond surface, while Figure 5d shows the histogram plot for the best-fit *z*_0_ values over the entire diamond surface. We measured an average *W*_adh_ values of 80 ± 20 mJ/m^2^ and an average *z*_0_ value of 0.6 ± 0.2 nm, where the error represents the standard deviation of the mean values determined. Qualitatively comparable observations were made with the other AFM tips tested in these series of measurements. There is limited literature pertaining to the *W*_adh_ and *z*_0_ values for a silicon/SiO*_x_*–diamond system [47,54]. Moore and Houston calculated a *W*_adh_ value between 200 and 400 mJ/m^2^, while Jacobs et al. calculated a *W*_adh_ value of 660 mJ/m^2^ and a *z*_0_ value of 0.25 nm. We observe that the measured *W*_adh_ values in this study are one magnitude lower than those in either of these studies. However, it is important to note that the forces measured in the two aforementioned studies happened with direct static contact between the tip and substrate, thus resulting in significantly smaller tip–sample separation distances. It is possible that the forces measured in these studies are indicative of other types of dominating forces such as covalent interactions that may not play such a significant role when using FM-AFM.

**Figure 6:**
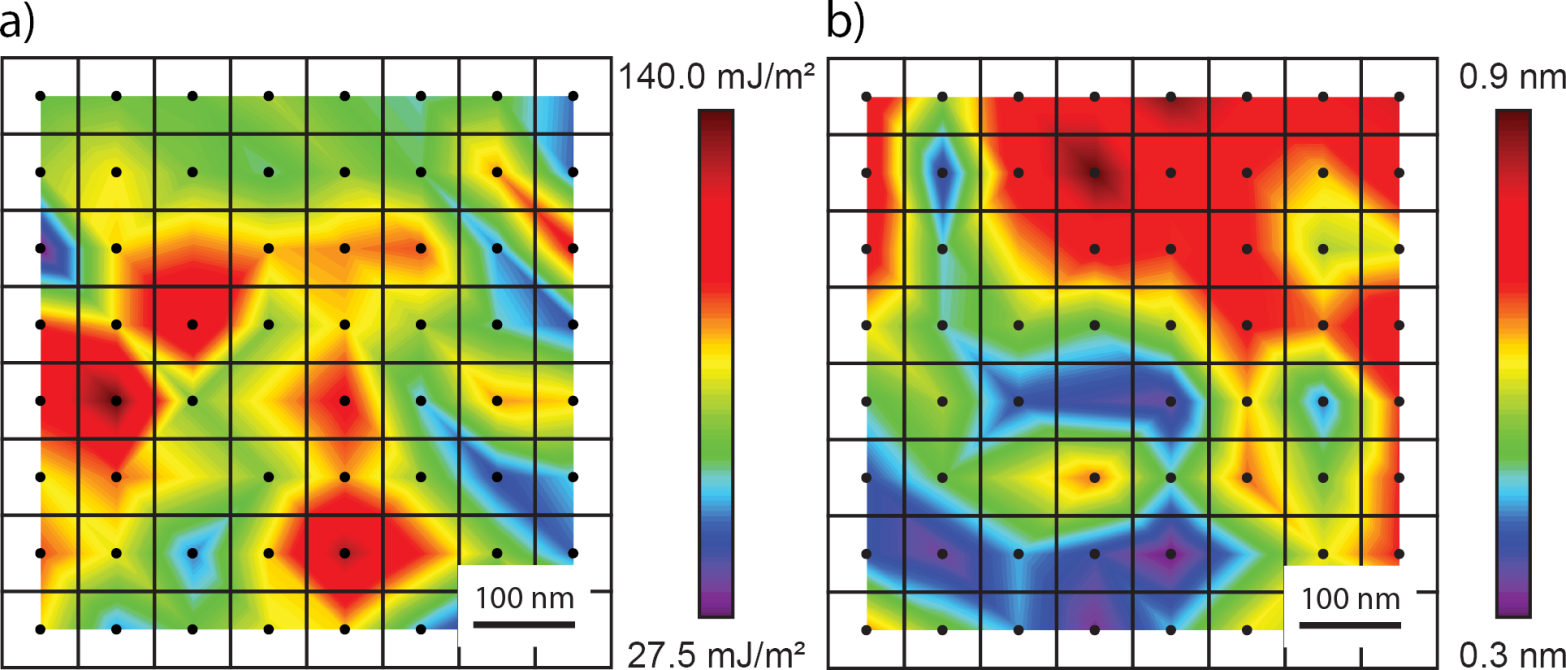
a) Averaged best-fit *W*_adh_ value over a 500 nm × 500 nm scan area of the diamond surface. b) Averaged best-fit *z*_0_ value over a 500 nm × 500 nm scan area of the diamond surface. There are four positions in this scan area for which all frequency curves were disregarded as the extracted *F*(*z*) curves contained multiple local force minima.

To obtain a better understanding of the variation in the measured values for *W*_adh_ and *z*_0_ over the diamond surface, as well as deviations of measured values from those reported in previous literature, the values for the *W*_adh_ and *z*_0_ in our experiments were plotted spatially. Specifically, Figure 6a and Figure 6b show the averaged best-fit *W*_adh_ and *z*_0_ values, respectively, over a 500 × 500 nm^2^ scan area. One hypothesis for these variations in *W*_adh_ and *z*_0_ is that localized roughness of the diamond surface significantly affects the adhesion interactions between a material pair. In a recent investigation, matched TEM experiments and MD simulations showed that a probe with a tip RMS roughness of 0.15 nm, decreased *W*_adh_ by approximately 50 percent over a smooth tip [12]. In our case, the localized roughness analysis performed in Figure 3 showed no significant correlation with the localized *W*_adh_ or *z*_0_ values. This result may have arisen from the low grid resolution when performing Δ*f*–*d* curve measurements, making direct comparisons of surface roughness and acquired values *W*_adh_ and *z*_0_ difficult. Thus, future studies should be performed over similar diamond surfaces with higher grid resolution in order to determine the experimental significance of surface roughness for the determination of adhesive parameters using FM-AFM. Additionally, one could use a material such as atomically flat highly oriented pyrolytic graphite (HOPG) or a KBr crystal to negate the effects of varying scales of roughness, which [12] suggests should be a contributing factor.

Qualitatively, there is a general mismatch in behavior when comparing the majority of experimental results with the LJ *F*(*z*) curves. In particular, the LJ *F*(*z*) curves generally predict higher attractive forces at larger tip–sample separation distances when compared with the experimental results. Additionally, the LJ *F*(*z*) curves overestimate the repulsive forces for smaller tip–sample separation distances. The attractive term in the 6-12 LJ pair potential has a *z*^−6^ dependency that can be mathematically derived to describe long-range van der Waals force interactions [55]. However, the repulsive term in the 6-12 LJ pair potential has a *z*^−12^ dependency that is empirical and computationally simple [32,56]. While it is known that the repulsive term in the 6-12 LJ pair potential is an over-simplification, we demonstrate that it is significantly misleading for the near-contact behavior of silicon oxide and diamond.

There are several factors that may have led to this discrepancy. First, the LJ potential, which is extremely widely used in contact mechanics and even in atomistic simulations of solid interfaces, does not accurately capture the interactions between silicon and diamond surfaces. More specifically, if we observe the mismatch in the repulsive force component, assuming that the attractive force component follows a *z*^−3^ dependency, we see that the LJ *F*(*z*) curves underestimate the repulsive forces at larger tip–sample separation distances and overestimate the repulsive forces at smaller tip–sample separation distances. These contrasting behaviors suggest that the repulsive term in the 6-12 LJ pair potential does not properly characterize the repulsive forces experienced in a silicon oxide–carbon system. This is somewhat expected, as several experimental studies have shown this repulsive *z*-exponent to vary between 9 and 14 [32,57]. Furthermore, some studies have suggested that an exponential term has more basis as a repulsive term in atomic pair potentials [32,58–59]. While the current methodology, using the 6-12 LJ pair potential, was not seen to accurately model the interaction force behavior in a silicon oxide–carbon system, the same methodology can be applied using a variety of pair potentials as a method for determining appropriate pair potential forms for any material system.

Second, general deviations between the experimental and LJ *F*(*z*) curves could relate to the experimental data that would result in an ill-posed conversion of the Δ*f*–*d* curves to *F*(*z*) curves [33]. Sader et al. showed that small changes in the frequency measurement due to noise or step-like interaction force changes can significantly change an experimentally extracted *F*(*z*) curve converted from a measured Δ*f*–*d* curve. This factor should be considered minimal due to relatively large oscillation amplitudes used in the present study. Sader showed that this ill-posed nature did not occur when oscillation amplitudes were larger than *z*_inf_/2, where *z*_inf_ is the distance corresponding to the inflection point of the governing force law acting between the tip and the substrate. In this study, *z*_inf_/2 is of the order of *z*_0_ and therefore always at least one magnitude lower than our oscillation amplitudes. Dagdeviren and Schwarz further showed that these effects proved negligible in vacuum-based experiments when oscillation amplitudes are greater than 1 nm [60]. Regardless, further investigation into the amplitude configuration of the Δ*f*–*d* curves should be performed to confirm that the force conversion is not ill-posed.

Third, static compensation of electrostatic forces was performed to account for surface potential differences between the tip and the sample surface. However, several studies have shown that there is a *z*-dependency of the required electrostatic compensation [61–62]. This factor would result in changes in the electrostatic interaction forces between the tip and sample as a function of the tip–sample separation distance. Further investigation into the significance of this dependency should be performed in the context of the silicon oxide–carbon material system.

Lastly, local variations in surface termination or bonding configuration of the carbon atoms on the sample surface could result in significant changes in the measured *W*_adh_ values recorded across the diamond surface. It has been previously shown that changes in surface termination, such as hydrogen- and oxygen-termination, as well as chemical aging of the diamond surface can lead to significant variations in surface energy [63]. Furthermore, a loss of hydrogen termination of the diamond surface could result in CH*_x_* defects [64–65] as well C–C dimer surface reconstruction [66]. Therefore, a chemical analysis of the diamond surface or an analysis of a surface less susceptible to spatial chemical variation under UHV conditions is required to further explore this last limitation.

## Conclusion

We have developed a novel experimental method to determine the precise interaction force curve using a combined TEM-AFM method. In particular, the uniqueness of this technique lies in the ability to use the experimentally measured interaction force curves combined with the tip shape to extract the fundamental physical interactions between the tip and the surface. Our methodology relies on the availability of a TEM to characterize the tip limits thus limiting its broad applicability. However, it is possible that other tip characterization methods, such as blind tip reconstruction, could be applied instead. Furthermore, the results highlight a wider-ranging issue: Improved methods for modeling adhesive contacts beyond those based on LJ potentials are necessary for improving the fidelity of such models and thus for a better understanding of the underlying physical mechanisms that occur when asperities come into contact. First, our methodology can serve to deconvolve material pair potential parameters from FM-AFM measurements between a tip, with complex geometry, and a flat substrate. Second, it can serve as an experimental tool to qualitatively validate appropriate pair potentials for a given material system. In this study, Δ*f*–*d* curves were acquired using FM-AFM on a diamond surface with a silicon AFM probe with its native oxide. With these measurements, experimental *F*(*z*) curves were analytically determined using the method shown in [45] and compared to theoretical LJ *F*(*z*) curves obtained by integrating the 6-12 LJ pair potential over the geometry of the experimental AFM tip apex. From these comparisons, the best fit *W*_adh_ and *z*_0_ values were determined using a least squares regression. Values for the *W*_adh_ and *z*_0_ parameters were calculated to be 80 ± 20 mJ/m^2^ and 0.6 ± 0.2 nm, respectively. Qualitatively, the 6-12 LJ pair potential generally overestimated the repulsive interaction force at smaller tip–sample separation distances and overestimated the attractive interaction force at larger tip–sample separation distances. The overestimation suggests that the 6-12 LJ pair potential may not be suitable to model the interaction behavior that occurs in the silicon–carbon system. These observations warrant future investigations into the effects of different interaction potential modeling, surface roughness, ill-posed Δ*f*–*d* curve nature and *z*-dependency in the contact potential difference of the AFM tip and sample, with respect to the determination of adhesive parameters using FM-AFM. Further, a detailed examination on the impact of small changes in tip shape on the generated potential is warranted, given the specificity of the potential to surface roughness, tip shape, and material pair.
